# Maxillary Expansion in the Management of Obstructive Sleep Apnea: A Comprehensive Review

**DOI:** 10.3390/dj13090410

**Published:** 2025-09-05

**Authors:** Roqaya Alrumaih, Ali Alterki, Mohammad Qali

**Affiliations:** 1 Ministry of Health, Kuwait City 13001, Kuwait; ralrumaih99@gmail.com (R.A.); alialterki@gmail.com (A.A.); 2 Department of Surgical Sciences, College of Dentistry, Health Sciences Center, Kuwait University, Kuwait City 13060, Kuwait

**Keywords:** maxillary expansion, obstructive sleep apnea, RME, MARPE, SARPE, DOME

## Abstract

Obstructive sleep apnea (OSA) is a prevalent sleep disorder characterized by partial or complete blockage of the upper airway during sleep, leading to disrupted airflow and fragmented sleep. Maxillary expansion has emerged as a promising treatment option, as widening the maxilla can increase nasal airway volume and improve airflow. The treatment options for maxillary expansion include rapid maxillary expansion (RME) for pediatric patients, mini-screw-assisted rapid palatal expansion  (MARPE) for adolescents and adults, surgically assisted rapid palatal expansion (SARPE) for skeletally mature individuals, and distraction osteogenesis maxillary expansion (DOME) for adults with severe maxillary constriction and nasal obstruction.  This comprehensive review explores the role of maxillary expansion in the management of OSA, examining its clinical applications and potential effectiveness as a therapeutic intervention.

## 1. Introduction

Obstructive Sleep Apnea (OSA) is a sleep disorder characterized by repeated episodes of partial or complete upper airway obstruction during sleep, leading to disrupted breathing, intermittent hypoxia, and fragmented sleep [1]. OSA is a prevalent disease that is spread worldwide. Epidemiological modeling by Benjafield et al. [2] estimated that up to 936 million adults aged 30–69 years worldwide may be affected by OSA, with approximately 425 million experiencing moderate to severe disease. While prevalence estimates vary between regions due to limited national data, this study seems to be the most comprehensive attempt to quantify the global burden of OSA to date. Although global prevalence estimates are limited by the scarcity of country-specific studies, the findings from the paper provide a critical baseline for understanding the magnitude and urgency of this growing public health concern. It has significant public health implications due to its association with hypertension, cardiovascular disease, type II diabetes, psychiatric disorders, glaucoma, renal dysfunction, and increased accident risk due to insufficient sleep [3].

Beyond medical comorbidities, OSA has a profound negative impact on quality of life (QoL), often manifesting as fatigue, excessive daytime sleepiness, impaired attention, emotional distress, and impaired psychological functioning [3,4]. In addition to clinical burden, OSA poses a significant economic strain, with the annual cost of diagnosing and treating OSA in the United States alone estimated at $12.4 billion US dollars, and global cost estimates remaining uncertain due to limited prevalence data [2]. Despite its high burden, an estimated 80–90% of individuals with OSA remain undiagnosed [5]. This contributes to delays in treatment and persistent comorbid risks. Furthermore, OSA is an independent risk factor for all-cause mortality, with mortality risk rising alongside disease severity [5]. Therefore, understanding the anatomical and physiological contributors to OSA, such as maxillary constriction, is essential for developing targeted, cost-effective interventions that can address both the functional airway compromise and systemic consequences.

With different treatments available for OSA, maxillary expansion has sparked an interesting role for its ability to tackle the underlying issues of airway blockage. Typically employed in orthodontics to fix dental and skeletal problems, maxillary expansion has demonstrated potential in enhancing upper airway measurements, especially in individuals with narrow palates and impaired nasal breathing [6]. Transverse maxillary deficiency with a high-arched palate is linked to narrow nasal passages, increased nasal airflow resistance, and reduced intraoral volume for the tongue [7]. These anatomical features compromise upper airway patency and contribute to one of the recognized craniofacial phenotypes of OSA [7]. Maxillary expansion directly targets this anatomical restriction by widening the nasal cavity and increasing space in the oral cavity, allowing the tongue to sit in a more anterior and superior position [8]. This structural change reduces airway resistance and helps prevent posterior airway collapse during sleep [8]. Thus, by improving maxillary width, expansion directly enhances upper airway volume and function, offering a structural and therapeutic benefit for appropriately selected patients with OSA, particularly those with a maxillary deficiency, while being less suitable for those whose OSA is unrelated to craniofacial anatomy.

Maxillary expanders are divided into five categories and are chosen according to the age and growth of the patient and the maxillary discrepancy. Conventional rapid maxillary expansion (RME) uses a fixed appliance to separate the midpalatal suture in growing children and adolescents and is accompanied by substantial skeletal alterations. It is the first-line treatment for children and adolescents because their sutures are not fully fused, and they have the potential to undergo significant skeletal changes. Mini-screw-assisted rapid palatal expansion (MARPE) is recommended for older adolescents and adults, and it uses skeletal anchorage with conventional orthodontic appliances to obtain a good expansion in patients with stiff sutures. For the completely mature skeletal patients, surgically assisted rapid palatal expansion (SARPE) includes surgical management to enforce the midpalatal suture for expansion with minimal dental tipping. Finally, distraction osteogenesis maxillary expansion (DOME) is a relatively new method that is mainly employed for the treatment of severe OSA in adults with narrow maxillae. DOME is a more recent modality that combines the concepts of distraction osteogenesis with maxillary expansion to achieve skeletal and nasal airway expansion.

While numerous studies have explored the effects of individual maxillary expansion techniques—such as RME, MARPE, SARPE, and DOME—on OSA, the existing literature remains largely fragmented, with each technique often reported in isolation and across varied study designs and patient groups. There is currently no consolidated review that comprehensively synthesizes the available evidence on all four modalities in the context of OSA. This review explicitly addresses that gap by providing a unified overview of the indications, patient selection, mechanism of action, clinical outcomes, evidence, limitations, and considerations associated with each technique. It aims to make the available knowledge more accessible and clinically actionable by clearly outlining the shared principles and distinct features of these approaches. By doing so, it supports clinicians and researchers in identifying the most appropriate expansion modality for different patients and clinical scenarios. Several gaps in the existing literature justify the need for this review. First, although multiple studies have reported anatomical changes following maxillary expansion, the evidence is fragmented and inconsistent regarding its effectiveness in improving clinical outcomes for OSA. Second, there is no clear consensus on how to match expansion techniques to patient-specific factors such as age and skeletal maturity. Third, while newer approaches such as MARPE and DOME are increasingly used, their long-term outcomes and comparative advantages over conventional methods remain insufficiently explored. A comprehensive synthesis of the available evidence is needed to guide interdisciplinary decision-making between orthodontists, sleep physicians, and surgeons. Therefore, the objective of this review is to evaluate and compare the current evidence on the effectiveness of rapid maxillary expansion (RME), mini-screw-assisted rapid palatal expansion (MARPE), surgically assisted rapid palatal expansion (SARPE), and distraction osteogenesis maxillary expansion (DOME) in the treatment of OSA, particularly in patients with transverse maxillary deficiency. This review seeks to answer the clinical question: To what extent do various maxillary expansion techniques improve airway dimensions and reduce symptoms in patients with obstructive sleep apnea?

## 2. Methods

### 2.1. Literature Search Strategy

This review is based on a comprehensive literature search conducted primarily through the PubMed database. Additional sources, including the Cochrane Library and Google Scholar, were also explored to ensure broad coverage. The search included studies evaluating the effects of maxillary expansion on OSA, as well as studies assessing skeletal and dental changes, mechanism of action, clinical indications, patient selection, side effects, and limitations of the following techniques: RME, SARPE, MARPE, and DOME.

Search terms included combinations of “rapid maxillary expansion”, “rapid palatal expansion”, “surgically assisted rapid palatal expansion”, ”miniscrew-assisted rapid palatal expansion”, “micro-implant assisted rapid palatal expansion,” “distraction osteogenesis maxillary expansion”, “RME”, “SARPE”, ”MARPE”, “DOME”, “obstructive sleep apnea”, “obstructive sleep apnoea”, “OSA”, “airway volume”, “transverse maxillary deficiency”, “nasal resistance”, “skeletal effects”, and “dental expansion”. These keywords were combined to capture a wide range of relevant literature.

No age restrictions were applied to the patient populations. However, only studies published within the last 10 years were included. Articles were excluded if they were older than 10 years, not in English, or did not provide clinically relevant or anatomical data related to maxillary expansion.

### 2.2. Study Quality Assessment

As this is a comprehensive review, no formal risk-of-bias tool was applied. However, a selective approach was used to ensure the inclusion of studies with clinical relevance and adequate methodological transparency. Priority was given to articles that provided detailed descriptions of study design, inclusion/exclusion criteria, intervention protocols, and outcome measures.

Included studies covered a range of study types—including systematic reviews, randomized control trials, prospective, and retrospective—and addressed key aspects such as skeletal and dental effects, airway dimensional changes, apnea–hypopnea index (AHI) reduction, patient selection, clinical indications, side effects, and treatment limitations. Peer-reviewed publications were prioritized to enhance the reliability of findings, although inclusion was not strictly limited to them. This approach allowed for a broad yet focused synthesis of the literature, offering clinically relevant insights while acknowledging the heterogeneity of available evidence.

## 3. Rapid Maxillary Expansion

RME refers to the technique used to widen the maxilla by separating the mid-palatal suture, while the rapid palatal expander (RPE) is the appliance used to deliver the forces necessary for this separation.

### 3.1. Indications and Patient Selection

RME is recommended for patients with maxillary constriction that may lead to crowding, posterior crossbites, and other problems associated with narrow upper jaws. Patients with a transverse maxillary deficiency, which is often diagnosed through clinical examinations, cephalometrics, cone-beam computed tomography (CBCT), and model analysis, may benefit from RME. Children and adolescents who have not completed their growth are usually the most suitable candidates for RME because their bones are still soft and easily influenced by the orthopedic forces [9]. Adult patients can also undergo RME, but the procedure may not be as effective as in children and adolescents since the mid-palatal suture is often fused in adults.

### 3.2. The Mechanism of Action

RME is achieved by the application of controlled lateral forces to the maxilla to open the mid-palatal suture. This separation enables the maxilla to expand and create more space for the proper alignment of the teeth and the correction of the malocclusion. The expansion of the maxilla also influences the zygomatic bones and thus increases the width of the upper dental arch. Besides skeletal expansion, RME also stimulates bone formation in the mid-palatal suture area, which helps in the stability of the expansion [10].

### 3.3. Clinical Outcomes and Evidence

**Figure 1:**
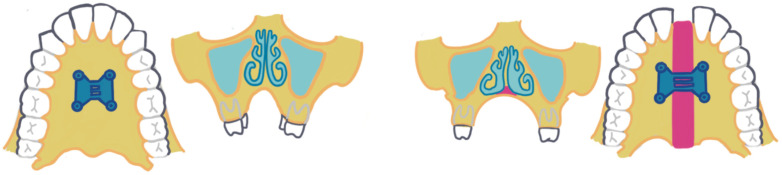
Sutural opening and the difference in the palate shape before and after expansion.

There are many studies that have shown that RME is an effective treatment for transverse maxillary deficiencies and other malocclusions [11,12,13]. The results of the treatment usually include an increase in the width of the maxilla, proper alignment of the dental arches, and the elimination of crossbites. Patients who are subjected to RME may also gain benefits in breathing, speech, and facial aesthetics. Long-term studies have shown that the effects of RME are usually stable, but some degree of relapse may occur if no proper retention protocols are followed [14]. In general, the evidence suggests that RME is a successful orthodontic treatment for patients with maxillary constriction [6]. Tsolakis and Kolokitha [15] reported that rapid maxillary expansion results in a statistically significant enlargement of both the volumetric dimensions and the minimal cross-sectional area of the nasal airway. It was also shown that RPE in growing patients significantly increases nasal cavity dimensions, with mid-palatal suture opening following a triangular expansion pattern [16]. Figure 1 showing a case with RME before, during, and after completion of RME with orthodontics.

### 3.4. Limitations and Considerations

Although RME is quite effective, there are some limitations and considerations that should be considered. Patients who are undergoing RME may have some side effects, such as pain, discomfort, and difficulty speaking or eating during the expansion phase. It is therefore important that patients adhere to the treatment protocol for successful outcomes, including the use of a fixed or removable retainer after expansion. In some cases, RME may not fully address complex skeletal discrepancies that may need further orthodontic treatment, such as orthognathic surgery. Thus, it is important to ensure that the patient is properly selected, and the limitations and special considerations are taken care of, along with a well-laid-out treatment plan to achieve the best results with RME. Overall, RME is a successful and reliable way of addressing maxillary constriction and enhancing the overall oral health and aesthetics [17].

## 4. Surgically Assisted Rapid Palatal Expansion

SARPE represents a specific combination of orthopedic and orthodontic techniques used to treat transverse maxillary deficiencies after patients complete their skeletal development, typically after adolescence. Patients with fused mid-palatal sutures and adjacent articulations fail to respond to standard RME methods. SARPE achieves true skeletal maxillary widening through the combination of selective osteotomies with mechanical expansion forces for patients who need surgical intervention [18].

### 4.1. Indications for Surgical Intervention

The main purpose of SARPE treatment is to help skeletally mature patients who have transverse maxillary deficiency through symptoms that include unilateral or bilateral posterior crossbite, arch constriction, and severe dental crowding. The procedure serves several purposes, including expanding the orthodontic treatment space for alignment, preparing patients for orthognathic surgery, and enhancing smile esthetics and functional balance. Dental compensation without actual skeletal changes occurs in adult patients during nonsurgical expansion attempts, which leads to the necessity of surgical intervention for stable and predictable outcomes.

### 4.2. Mechanism of Action

The procedure starts with surgical weakening of resistance areas such as the mid-palatal suture and lateral walls of the nasal cavity, as well as the pterygoid plates when fusion is severe [19]. The expansion process starts after surgical release when the palatal appliance, including the Hyrax screw or Haas expander, undergoes gradual activation to separate the maxilla’s two halves. The mechanical pressure enables new bone development in the expanded area, which leads to stable skeletal growth of the transverse dimension instead of dental movement that occurs during traditional adult orthodontic procedures.

### 4.3. Clinical Outcomes and Evidence

Multiple clinical studies and case series research have proven that SARPE achieves dependable transverse skeletal expansion while producing minimal dental complications. Studies have documented that SARPE treatment produces expanded nasal airway spaces [20]. Research data indicates that SARPE skeletal results maintain stability over time when proper retention methods are implemented [11]. Research findings show better QoL results after treatment, particularly concerning breathing abilities [20]. SARPE outperforms adult non-surgical expansion because it produces more beneficial effects on periodontal health through reduced excessive buccal tipping and alveolar bone bending [21].

### 4.4. Limitations and Considerations

SARPE remains a highly effective procedure, yet it has several limitations that should be considered. Surgical intervention for SARPE carries risks, including infection along with bleeding, nerve injury, discomfort, relapse, and the possibility of infection [22]. The treatment outcome can be negative if patients do not follow their post-expansion retention instructions or if they fail to comply with their treatment plan. The surgical process demands precise planning because the goal is to prevent harm to the nasal mucosa tissues and tooth roots. The success of SARPE heavily depends on psychological factors, as well as the patient’s compliance and the ability of orthodontists and surgeons to work together as a team. The achievement of optimal results and the reduction in complications requires accurate diagnosis together with proper case selection and complete patient education [22].

## 5. Mini-Screw-Assisted Rapid Palatal Expansion

### 5.1. Indications

#### 5.1.1. Advantages over RME

With the implementation of temporary anchorage devices (TADs), MARPE enables greater skeletal expansion without the risk of root resorption, periodontal defects, dentoalveolar tipping, or relapse [8].

#### 5.1.2. Patient Selection

MARPE corrects an underdeveloped and abnormally narrow maxilla in adults, solely through force exertion onto the basal bones, with no osteotomies required [23].

##### Influence of Suture Ossification Stage in Young Adults

MARPE can be used in adults over 18 years old with a transverse maxillary deficiency [23]. After adolescence, the mid-palatal suture progressively ossifies and interdigitates with age [23]. In a prospective study with patients above 18 years of age and a mean age of 24.5, the patients presented with varying degrees of ossification of the mid-palatal suture and were grouped based on the degree of ossification and whether the fusion was completed in the palatine bone or was more advanced with fusion in the anterior maxilla [23]. The results of this study revealed that MARPE resulted in a statistically significant improvement in upper airway volumetric parameters, irrespective of the degree of ossification of the mid-palatal suture [23]. There was an average increase of approximately 14% in the volume of the upper airway in both groups [23]. The results suggest that MARPE has consistent airway benefits in young adults, irrespective of ossification stage.

### 5.2. MARPE Step by Step

#### 5.2.1. Appliance Fabrication

Orthodontic bands are fitted onto the upper first permanent molars. Alginate impressions are taken, and the bands are transferred onto the impression, which is then sent to the lab for pouring in plaster. The resulting plaster cast aids in band soldering and wire bending.

#### 5.2.2. Appliance Fitting and Mini-Screw Insertion

The expander is then fitted intraorally and cemented with glass ionomer [23]. To facilitate oral hygiene, a mucosal clearance gap of 1.5–2 mm is maintained to allow access for cleaning and tube placement [24]. Under local anesthesia, the mini-implants are inserted into pre-perforated palatal bone using a bur [23]. The typical configuration includes 4–6 mini-implants, 1.8 mm in diameter and 9–13 mm in length, selected based on palatal bone thickness.

#### 5.2.3. Activation Protocol

Initial activation is 0.5 to 1 mm per day. Once an upper midline diastema is observed, the protocol may be reduced to 0.25–0.5 mm/day. Some clinicians prefer slower activation, adjusting the appliance once daily, with each activation involving a quarter turn of the screw, equivalent to 0.2 mm of expansion for more controlled movement [25]. The expansion protocol may differ among clinicians. The duration of active expansion is typically 4–6 weeks, depending on the degree of expansion needed [26].

#### 5.2.4. Monitoring and Maintenance

Regular follow-up is essential to monitor progress, reinforce oral hygiene, and ensure the correct activation protocol is maintained.

#### 5.2.5. Retention Phase

A six-month retention period allows for bone ossification and stability while the expander remains in place [3]. This phase aids in preventing relapse and typically lasts 4–6 months [26]. During this period, active orthodontic movement should be deferred.

### 5.3. Mechanism of Action

With MARPE, mini-implants are placed directly into the maxillary palatal bone to secure the expansion appliance, avoiding dental anchorage [27]. This direct skeletal anchorage enables superior orthopedic expansion of the maxilla in comparison to conventional RME, which uses dental support [28]. Undesirable tooth movement and dentoalveolar effects are avoided [27]. In addition, greater nasal expansion is attained in comparison to the conventional RME [27]. Activation of the appliance results in mid-palatal suture opening and lateral displacement of the nasal walls, resulting in nasal floor expansion [28]. This reduces nasal resistance, which is a known predisposing factor for upper airway obstruction, and it also contributes to OSA improvement [28,29]. The expansion of the nasal cavity and maxillary arch enhances airway patency and improves breathing during sleep, as supported by recent findings [30]. Collectively, these biomechanical effects establish MARPE as a promising non-surgical alternative for improving airway function in patients with OSA.

#### 5.3.1. Airway Volume Changes in Adults

**Figure 2:**
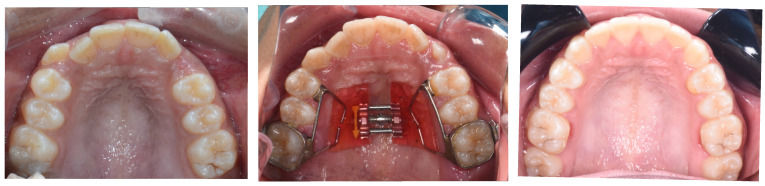
A case with RME before, during, and after completion of RME with orthodontics.

In a study by Li et al. [31], MARPE led to a 14.1% increase in nasopharyngeal airway volume and a 16.2% increase in nasal cavity volume in adults over 18. This suggests MARPE may significantly improve nasal breathing by enlarging upper airway structures that directly affect ventilatory function. However, no significant volumetric changes were observed in the retroglossal, retropalatal, or hypopharyngeal regions [31]. This indicates that MARPE’s impact is largely localized to the upper airway, with a limited effect on lower pharyngeal regions involved in airway collapse. Therefore, it could be inferred that MARPE’s therapeutic benefit in OSA is likely most pronounced in patients with upper airway obstruction localized to the nasopharyngeal airway, whereas its impact on deeper pharyngeal segments may require adjunctive interventions to achieve meaningful outcomes. Figure 2 showing Sutural opening and the difference in the palate shape before and after expansion.

#### 5.3.2. Systematic Review on Upper Airway Volume

Li et al. [32] conducted a systematic review and meta-analysis evaluating the effects of MARPE on the volume of the upper airway. The range of increase in the nasal cavity volume after expansion was from 9.21% to 22.73%; meanwhile, after retention, the range of increase was from 10.1% to 77.2%, and all measurements demonstrated statistical significance [32]. This volume increase contributes to improved nasal breathing, which is especially beneficial in OSA patients who typically experience airway obstruction. This also suggests that the airway benefits of MARPE take time to manifest, which reinforces the importance of retention protocols in managing OSA patients. The nasopharyngeal volume also increased, with a range from 6.4% to 19.99% post-expansion, and from 8.48% to 47.9% post-retention [32]. However, the nasopharyngeal volume showed no significant changes immediately after expansion, but changes after retention demonstrated clinically significant results [32]. Thus, immediate changes post-expansion may not be significant. However, retention leads to a significant increase in the nasopharynx volume. This delayed skeletal modeling may contribute to OSA improvement by enhancing upper airway patency and reducing pharyngeal collapse during sleep. The volume of the oropharynx demonstrated a significant increase post-expansion, but after retention, there were no significant changes [32]. An increase in oropharynx volume is seen immediately after expansion, suggesting that MARPE may positively affect the oropharyngeal airway space during and shortly after treatment. This increase was not sustained after the retention period, which contrasts with nasal and nasopharyngeal volumes. Therefore, MARPE may offer short-term benefits for OSA in the oropharynx, but long-term improvements may not persist with MARPE alone. None of the included studies in the review has investigated changes in the palatopharynx volume following expansion [32]. However, three studies assessed palatopharynx volume after the retention period, and a significant increase was not detected [32]. This suggests that MARPE does not produce a significant increase in this region even after retention. Hence, this region likely plays a minimal role in MARPE’s contribution to OSA improvements compared to the nasal cavity and nasopharynx, which showed consistent and statistically significant volume gains. Similarly, no study reported post-expansion changes in the glossopharynx, though three studies evaluated this region after retention and found no significant volumetric changes, with moderate heterogeneity [32]. For the hypopharynx, no studies assessed immediate post-expansion changes, and the four studies evaluating this region after retention also reported no statistically significant increase [32]. This pattern suggests a limited and inconsistent impact of MARPE on the lower pharyngeal airway regions, possibly due to their anatomical positioning and reduced involvement in transverse skeletal expansion. Therefore, MARPE may cause significant long-term increases in the volume of the nasopharynx and nasal cavity, while no significant changes were observed in the oropharynx, hypopharynx, glossopharynx, and palatopharynx.

#### 5.3.3. Immediate vs. Long-Term Airway Changes (11–15-Year-Olds)

Immediate post-expansion changes were observed in a study by Mehta et al. [28], demonstrating that MARPE led to statistically significant improvements (*p* < 0.05) in several airway parameters, including a 14.4% increase in nasal cavity volume, 21.8% in nasopharyngeal volume, 22.7% in nasopharyngeal area, 19.2% in oropharyngeal volume, 20.5% in total airway volume, 8.1% in total airway area, and 10.4% in total palatal width. Following an average of 2 years and 8 months post-MARPE, during which patients underwent orthodontic treatment, another CBCT was taken to assess long-term outcomes [28]. Although some of the improvements could be partially attributed to natural craniofacial growth since the patients were between 11 and 15 years of age, the MARPE group demonstrated greater increases in nasopharyngeal parameters compared to RPE and controls, suggesting a true treatment effect [28]. However, it is worth noting that at the long-term follow-up, oropharyngeal volume, total airway volume, and total airway area were greater in the RPE and control groups [28]. This indicates that MARPE’s most substantial and lasting effects may be localized to the nasopharyngeal region. The findings revealed sustained improvements with a 29.6% increase in nasal cavity volume, 44.3% in nasopharyngeal volume, 41.9% in nasopharyngeal area, 35.6% in oropharyngeal volume, 38.5% in total airway volume, 17.4% in total airway area, and 9.3% in total palatal width [28]. These findings support the long-term efficacy of MARPE in enhancing upper airway dimensions, particularly in the nasopharyngeal region, and underscore its potential in managing pediatric OSA when applied during periods of active growth.

#### 5.3.4. Tooth-Borne vs. Tooth-Bone-Borne RME in Growing Patients

A randomized control trial with a 5-year follow-up on 52 patients compared tooth-borne (TB) RME and tooth-bone-borne (TBB) RME, with mean ages of 9.3 and 9.5 years, respectively [33]. The study demonstrated that TBB RME resulted in a significantly greater skeletal expansion of the mid-palatal suture [33]. Additionally, at the level of the nasal region, the skeletal expansion was significantly greater in the TBB RME group [33]. No differences between the genders were observed in terms of skeletal expansion [33]. Although the study referred to the appliance as TBB RME, its design and biomechanical function are comparable to MARPE, particularly in its skeletal anchorage via palatal mini-screws.

### 5.4. Clinical Outcomes and Evidence

#### Impact of MARPE on Adult OSA and QOL

MARPE expands the dimensions of both the oral and nasal cavities, reducing airflow resistance and contributing to improvement in OSA severity [3]. A prospective controlled trial revealed that MARPE significantly improved both structural and symptomatic markers in adult non-obese patients with OSA and a transverse maxillary deficiency [3]. The study demonstrated a mean AHI reduction from 28.75 to 11.45 events/h, representing a 65.3% decrease in OSA severity [3]. Among patients who achieved successful skeletal expansion, 78.5% experienced a 50% decline in AHI, and 35.7% reached an AHI of <5 events per hour post-treatment [3]. The patient with the most significant reduction in AHI had an improvement of 76.3%, while the lowest observed reduction was a 24.9% improvement [3]. A statistically significant improvement was observed in snoring duration, oxygen saturation, and apnea-associated bruxism, all noted solely in participants who underwent MARPE [3].

Mean oxygen saturations showed a statistically significant improvement from 91.92% to 94.32% in the intervention group [3]. The study showed that MARPE had a high success rate of 85% in adults [3]. The Epworth Sleepiness Scale (ESS) and Quebec Sleep Questionnaire (QSQ) improved as ESS decreased from 12.87 to 7.32 and total mean QSQ increased from 4.28 to 5.52, both of which were statistically significant [3]. These findings indicate that MARPE not only reduces OSA severity but also significantly improves sleep-related QoL in appropriately selected adult patients.

### 5.5. Limitations and Considerations

Complications and Side Effects

Complications associated with MARPE most commonly involve soft tissue irritation and inflammation, particularly around the mini-implants [3]. In one prospective study, 5 out of 32 participants experienced loss of the mini-implants as a result of mechanical instability, though this occurred during the retention phase and did not interfere with active expansion [3]. The same study reported no severe adverse effects such as oro-nasal fistula or bleeding of the nasal mucosa [3]. A few participants had to undergo a second expansion in which the initial jackscrew was removed, and another one was delivered the following day [3]. Labunet et al. [30] noted some minor soft tissue changes, such as papillary recession, but these were extrapolated from previous findings.

A retrospective study by Yoon et al. [26] found that 83.9% of patients experienced inflammation around the appliance, with 45% reporting tenderness or pain during and after expansion. Most patients who experienced pain did so on the day of the device instillation, which could be managed by 800 mg ibuprofen or 1000 mg paracetamol and resolved in the first two days for most patients [26]. Additionally, 10.5% experienced appliance breakage, and five teeth showed discoloration, two of which underwent root canal treatment, whilst the rest were monitored [26]. Asymmetric expansion >1 mm was noted in 47.8% of patients, which was managed conservatively with orthodontic treatment, and 27% had asymmetric expansion >2 mm [26]. Rare adverse events that could be associated with MARPE include short-term hearing loss, swelling, numbness, paresthesia, sinus infection, severe gag reflex, and discoloration of teeth, possibly indicating loss of vitality [26]. A single patient reported hypoesthesia of the infraorbital nerve, and spontaneous resolution occurred in 2 weeks [26]. The incorporation of mini-implants may create an environment that harbors microorganisms, increasing susceptibility to infection [26]. Thus, the positioning of the appliance should be carefully considered.

#### 5.5.1. Patient Related Factors

- Skeletal maturity, age, and suture ossification: Non-surgical expansion of the maxilla using orthopedic appliances relies on the mid-palatal maturation stage to assess treatment success. The predictability of suture separation declines with age, even when using TADs [27]. Obliteration of the mid-palatal suture varies amongst individuals; hence, determining the precise timing remains challenging [25]. In adults and late adolescents, the skeletal expansion associated with MARPE is limited due to mid-palatal suture interdigitation [31]. Previous research suggests that chronological age is a key factor influencing the success of maxillary expansion, as the maturation and fusion of the mid-palatal and surrounding circummaxillary sutures progress with age [25]. Maturation of the mid-palatal suture begins posteriorly, exhibiting individual variability in interdigitation, obliteration, and ossification patterns [25].
  The timing of complete ossification may not respond directly to the chronological age [25]. Hence, suture maturation could be an alternative method to assess eligibility to achieve orthopedic maxillary expansion [25]. Although histological examination remains the most definitive method for evaluation of midpalatal suture maturation, it is not feasible in living patients [25]. Consequently, conventional computed tomography (CT) imaging falls short of providing a reliable microscopic-level assessment of suture obliteration, highlighting the challenge in accurately predicting the optimal timing for orthopedic expansion [25].
  A prospective study examining age-dependent effects on MARPE revealed that expansion could be achieved with greater ease in patients younger than 20 years in comparison to those aged 20 years and above [24]. As chronological age increases, skeletal-to-screw expansion ratio declines [24]. In this study, patients were divided into four groups based on age: early adolescents (11–14 years), late adolescents (15–19 years), young adults (20–24), and old adults (25–34.1 years), with suture separation success rates of 100%, 100%, 88.2%, and 85.7%, respectively [24]. In this study, two young males failed to demonstrate suture separation, which could be linked to the marked acceleration in suture obliteration after the age of 20 [24].
  In a retrospective study, 31% of patients did not achieve sufficient expansion; hence, a second attempt was required, in which 38% failed to achieve adequate expansion [26]. Thus, patients should be made aware of the potential of treatment failure. In this study, most failures were in patients above 25 years of age. Among patients with failures to achieve successful suture separation, males accounted for 67.7% of failures, while females accounted for 32.2% [26]. Additional treatment may be necessary if the suture split fails, and causes of failure may be due to failure of bone integration with micro-implants, osteoporosis, poor oral hygiene, tobacco consumption, excessive alcohol intake, and inadequate follow-up [26].
- Gender differences: A retrospective study examining suture separation success rates amongst the genders revealed a 61.05% success in males and a 94.17% success in females [25]. This suggests that gender may play a role in determining the success of suture separation and, ultimately, MARPEs’ success in achieving orthopedic expansion and managing OSA. The success of suture separation amongst genders, particularly in 21–25 year olds, was examined, and it was determined that the odds of suture separation were 22-fold greater in females than in males. The study revealed that participants older than 15 years experienced a decline in success rates, reaching a success rate of 53.85% in males and 92.59% in females, with an overall rate of 73.58% [25]. The success rate of females above 15 years exceeded that of males within every age subgroup [25]. In participants above 20 years of age, a statistically significant association was found between gender and successful suture separation [25]. The likelihood of suture non-separation with progressing age had a statistically significant increase in males, and success was not achieved in any male patient aged over 30 years [25]. This may be linked to stiffness of the skeletal complex and maturation in the craniofacial region [25]. Hence, treatment, especially for males, at a young age is associated with more successful outcomes, including skeletal expansion.
- Bone density: Patient-related factors such as palatal bone morphology may influence MARPE’s outcomes [30]. Increased palatal cortical bone thickness and flatter palatal planes have been associated with enhanced stability [30]. These findings underscore the need for careful case selection to maximize treatment success.
- Compliance: Patient compliance plays a critical role in the success of MARPE treatment. Consistent appliance activation according to the orthodontist’s instructions is essential to achieving timely and controlled suture separation. Poor compliance can result in asymmetrical expansion or undercorrection. Compliance with follow-up appointments is equally important, particularly during the retention phase, where premature appliance removal may increase the risk of relapse. Therefore, evaluating the patient’s willingness to adhere to treatment protocols should be an integral part of case selection and treatment planning.
- Oral Hygiene: It is challenging for patients to maintain good oral hygiene; therefore, the most common complication is inflammation around the appliance, which is managed with chlorhexidine rinses along with oral hygiene instructions if mild [26]. If the inflammation was severe, premature removal and antibiotics may be required [26]. The design of the appliance can be altered to prevent palatal tissue compression and facilitate better cleaning [26]. This shows the important role of good oral hygiene in achieving treatment success.

#### 5.5.2. Non-Patient Related Factors

**Figure 3:**
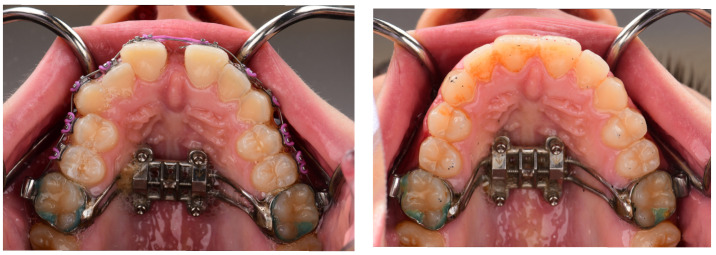
MARPE expander before and after diastema closure.

- Appliance design considerations: Numerous designs of the MARPE appliance exist. There are variations based on the jackscrews’ type, position, and the size of the mini-implants. The biomechanical design of the MARPE appliance influences its skeletal impact and subsequent airway response. MARPE can be categorized into three groups based on method of anchorage: bone-borne (BB), TBB, and tissue-bone-borne [24].
  The systematic review by Abu Arqub et al. [29] included studies with TBB and BB expanders. TBB appliances were associated with a statistically significant improvement in airflow of the nasal cavity and reduction in nasal resistance [29]. However, a major limitation of the systematic review is that it only examined three studies. Beyond the choice of anchorage method, the configuration and number of mini-implants may also influence skeletal expansion outcomes. Superior skeletal expansion is achieved by using a MARPE with four mini-screws in comparison to two mini-screws [24]. In adult patients with OSA, achieving mid-palatal suture separation is a prerequisite for skeletal expansion. While Choi et al. [34] showed that longer mini-screws resulted in greater skeletal expansion, the study also noted that longer screw length alone did not guarantee successful suture separation. Thus, successful suture separation is an important consideration in OSA patients who may require maximal skeletal change to achieve meaningful airway improvements.
  In addition to the anchorage method and the number of mini-implants, anatomical constraints must also be considered when selecting the appliance. Patients with a transverse deficiency greater than 8 mm often present a challenge when selecting an appropriate expander. They may have inadequate palatal width to place a maxillary expander greater than 8 mm; hence, a second expander might be required for patients with a narrow maxillary arch [24].
- Retention phase and long-term stability: One consideration when interpreting MARPE’s effects on airway volume is the role of the retention phase. Anéris et al. [23] reported that the upper airway volume continued to increase for up to 4 months post-expansion, suggesting that retention may play a key role in achieving full skeletal and soft tissue adaptation, regardless of suture ossification level. Therefore, appropriate retention protocols should be put in place to ensure maximum benefit. Figure 3. MARPE expander before and after diastema closure and retention of the MARPE to ensure maximum benefit.

- Variability in response: Expansion of the maxilla may improve AHI scores since widening of the nasal cavity may result in a reduction in airflow resistance [3]. The expansion also allows the tongue to sit in a more forward position, which helps stretch the soft palate muscles and enhances their tone and functional activity [3]. Due to the multifactorial nature of OSA, the therapeutic response to MARPE may vary significantly among individuals [3]. It is suggested that patients with greater pharyngeal obstructions, particularly at the level of the nasopharynx, are more likely to experience notable improvements following expansion [3]. These findings highlight the importance of individualized assessment in predicting outcomes, as anatomical site and severity of airway obstruction may significantly influence the therapeutic response to MARPE.

## 6. Distraction Osteogenesis Maxillary Expansion

### 6.1. Indications and Patient Selection

Careful patient selection is critical in determining which cases are suitable for DOME and predicting treatment success. A predisposing factor for the development of OSA is maxillofacial hypoplasia [35]. OSA presents with a characteristic phenotype, including a high arched palate and a constricted maxilla [7]. This presentation is often accompanied by posterior tongue displacement and elevated nasal airflow resistance, which further predisposes individuals to developing OSA [36]. DOME selection criteria include adults with OSA who present with a high-vaulted palate, minimal soft tissue excess, and a constricted nasal floor [7]. DOME offers expansion of the maxilla with less invasive osteotomy [35]. Achieving treatment success may require both resolution of the nasal obstruction and sufficient pharyngeal airway volume, as improved nasal airflow alone may not overcome upper airway collapsibility when airway volume is severely reduced [35]. Appropriate case selection plays a key role in achieving a successful outcome.

### 6.2. Step-by-Step Technique of DOME

#### 6.2.1. Expander Installation

A custom-fabricated maxillary expander is placed on the narrow palatal vault and is stabilized with mini-implants [27]. The mini-implants usually range from four to six and are placed on the mid-palatal suture [36]. This is performed by the orthodontist under local anesthetic [7].

#### 6.2.2. DOME Surgery

This step is performed by the surgeon under general anesthesia [36]. The surgeon performs a minimally invasive Le Fort 1 osteotomy, which does not require the pterygoid plates to be fractured [27]. Two incisions are made bilaterally at the maxillary mucogingival junction, approximately 1 cm above the maxillary incisor roots [7]. A vertical incision is created between the maxillary central incisor roots [7]. The primordial groove of the midpalate suture is deepened using a piezoelectric saw [37]. The midpalate suture is wedged open using straight osteotomes [36]. As the suture splits open, an upper midline diastema is immediately observed [27]. To ensure smooth and symmetric bilateral maxillary separation, the expander is activated to ensure effectiveness [36].

#### 6.2.3. Expander Activation

This step is performed by the patient after 5–7 days post-DOME surgery [27]. The expander is activated daily at a rate of 0.25 mm [36]. Expansion at the nasal floor of approximately 8–10 mm is attained within a month [37].

#### 6.2.4. Orthodontic Treatment

After completion of the expansion, approximately a month after the DOME surgery, the orthodontist then initiates orthodontic therapy. Fixed appliances or aligner therapy may be utilized, closing the gaps created by DOME and re-establishing occlusion [27].

#### 6.2.5. Bone Consolidation Phase

This phase can last for 3 months, during which the expander remains passively in situ [27]. However, to minimize relapse and ensure maximum bone fill, it is optimally left for 6–8 months [27].

### 6.3. Mechanism of Action

In adults, maxillary expansion could be achieved by the adjunctive use of expanders alongside minimally invasive surgery to re-establish the maxillary sutures [7]. Maxillary expanders are stabilized using mini-implants positioned over the mid-palatal suture, ensuring effective sutural separation and successful expansion, with adverse effects at the palatal arch and nasal floor being minimized [7]. The gap created by the osteotomy is healed through natural mechanisms of bone regeneration.

The anatomy of both the oropharyngeal and the maxillofacial regions is an essential factor contributing to the pathophysiology of OSA [8]. Transverse deficiency of the maxilla, coupled with a decreased oral cavity volume, is considered a contributing factor to the pathogenesis of OSA [27]. Thus, it is critical to understand the changes in this region after DOME treatment. A high arched palate with a narrow maxilla is linked to elevated nasal airway resistance [27]. Understanding the anatomical implications of maxillary deficiency is essential to appreciate the clinical rationale behind DOME.

#### 6.3.1. Structural Widening and Airflow Resistance

In addition to its skeletal effects, DOME produces notable structural changes within the nasal cavity and palatal region. Widening of the previously constricted hard palate, along with nasal floor widening after DOME, is linked to a decrease in nasal airflow velocity and a subsequent negative pressure reduction in the pharyngeal airway [35]. This interaction is associated with a decrease in AHI and the Oxygen Desaturation Index (ODI) [35]. DOME increases the volume of the nasal cavity while decreasing nasal airway resistance [35]. This translates into reduced OSA severity, and a study by Iwasaki et al. [35] hypothesized that the observed changes in airflow dynamics may underlie this improvement, although the precise mechanism remains unclear.

#### 6.3.2. Functional and Pressure-Based Changes

These structural changes give rise to meaningful improvements in airflow dynamics and pressure regulation. During DOME, the morphology of the palate is modified from a high arched configuration to a broader dome-like form, expanding the intraoral volume for the tongue, which enhances perceived nasal breathing [35].

Before treatment, subjects exhibited high airflow velocity in the nasal airway, which significantly decreased following the procedure [35]. Although airway velocity in the pharyngeal region remained consistent, a notable reduction in negative pressure was observed [35]. This suggests that the decreased nasal airflow velocity is linked to reduced negative pressure in the pharyngeal airway [35].

#### 6.3.3. Stability and Tongue Position

Structural improvements in the oral and nasal regions also influence tongue posture, further enhancing airway stability. These changes seem to contribute to improved upper airway stability during sleep and explain the observed reduction in OSA severity following DOME. Expansion post DOME permits a more anterior resting position for the tongue [36]. This seems to be a contributing factor to increasing airway patency and reducing obstruction. This helps maintain the stability of the posterior pharyngeal airway, preventing collapse during sleep [8].

#### 6.3.4. Radiographic Findings and Targeted Areas of Expansion

Radiographic evidence reinforces these findings and helps pinpoint the specific anatomical regions correlated with treatment success. In a study aimed at understanding the mechanism of action, improvements post-DOME were thought to be correlated with expansion of the internal nasal valve angle along with the surface area [36]. Previous findings have indicated that widening of the anterior third of the bony nasal passage, particularly in the region adjacent to the nasopalatine canal as visualized on CBCT, is associated with improvements in both subjective nasal obstruction and an objective reduction in AHI [8].

### 6.4. Clinical Outcomes and Evidence

#### 6.4.1. Improvements in AHI and Nasal Dimensions

In a cohort prospective study with a sample size of 20 patients, all subjects achieved separation of the mid-palatal suture along with expansion of both the maxilla and nasal floor [7]. The study demonstrated a substantial decline in nasal airflow resistance, AHI, ESS, nasal obstruction symptom evaluation (NOSE), and ODI [7]. The results suggest that DOME may play a role in reducing the functional burden of OSA and nasal obstruction. In this study, a pronounced elevation of the nasal floor width was evident at the anatomical sites aligned with the nasopalatine nerve and the palatal root of the first molar [7]. These anatomical improvements warrant further exploration into their impact on functional breathing parameters such as oxygen saturation and airflow velocity, as discussed in the following section.

#### 6.4.2. Airway Patency and Breathing Efficiency

Further improvements in mean AHI were reported in another retrospective study. Mean AHI decreased from 17.81 to 7.82 episodes per hour, accompanied by an increase in oxygen saturations from 88.15% to 90.90% [35]. This indicates improved gas exchange during sleep, which is vital for reducing OSA-associated fatigue. This study also examined airflow velocity, which decreased from 15.6 to 7.4 after DOME [35]. This indicates a reduction in resistance and improved airway patency, helping to relieve the symptoms of OSA [35]. Following DOME, the intermaxillary molar width expanded significantly from 34.04 to 41.6 mm [35]. Beyond objective metrics, subjective and sleep-related outcomes offer additional evidence on treatment efficacy, as highlighted below.

#### 6.4.3. Objective and Subjective Treatment Outcomes

Another retrospective analysis was conducted on a sample of 75 patients, which showed a substantial decrease in AHI from 17.7 to 8.2, alongside an increase in rapid eye movement (REM) sleep percentage from 14.4% to 22.7% [27]. Significant improvements were also observed in patient-reported outcomes. The NOSE score decreased from 10.9 to 3.3, indicating reduced nasal obstruction, while the ESS score dropped from 10.5 to 6.7, reflecting decreased daytime sleepiness [27]. Such improvements support the notion that DOME not only impacts anatomical structures but also leads to noticeable relief in patient-reported symptoms such as nasal blockage and daytime drowsiness.

#### 6.4.4. Consistency Across Studies

The consistent reduction in AHI observed across studies reinforces DOME’s effectiveness in improving breathing efficiency. For example, a cohort by Abdelwahab et al. [36] reported a decrease from 23.26 to 7.54 in mean AHI scores. This indicates a marked improvement in OSA severity. Additionally, the subjective measures of ESS and NOSE also improved from 10.3 to 6.53 and 10.87 to 3.27, respectively [36]. The replication of these results across multiple studies supports the reliability of DOME as a possible treatment modality for OSA.

#### 6.4.5. Anatomical Variability and Predictors of Efficacy

Deviation of the septum is a common cause of obstruction in the nasal cavity [37]. This may be treated with septoplasty, which has a success rate ranging from 43–85% in the initial septal correction procedure [37]. Persistent obstruction tends to manifest in patients with narrow high-arched palates [37]. In a study on patients with constricted vaulted palates and persistent obstruction of the nasal cavity even after septoplasty, the findings revealed the efficacy of DOME in treating such patients [37].

The mean NOSE score reduced, and the CT scans revealed nasal floor width expansion [37]. Collectively, these studies highlight the multifaceted benefits of DOME across structural, functional, and subjective domains, while also highlighting the need to tailor treatment to anatomical and functional characteristics of each patient. Therefore, the evidence supports DOME as an effective intervention for OSA, offering improvements not only in anatomical dimensions but also in subjective symptoms and sleep quality. These benefits appear reproducible, further validating their clinical utility.

#### 6.4.6. Measurement of Treatment Success

##### Objective Measures

1. AHI
  - Quantifies the number of apneic and hypopneic events per hour of sleep used to assess OSA severity [38].

2. Oxygen Saturations
  - Measures mean and minimum nocturnal oxygen levels to reflect hypoxemia severity.

3. ODI
  - Captures the frequency of oxygen desaturation events per hour of sleep, indicating intermittent hypoxemia [39].

4. Airflow Velocity
  - Evaluates the speed of airflow during respiration to assess improvements in airway patency after expansion.

5. Nasal Airflow Resistance
  - Nasal airflow resistance is the pressure difference between the nostrils and the back of the nasal cavity, expressed in pascals, divided by the airflow rate in millimeters per second [40]. It measures the resistance to airflow through the nasal passages, indicating how well expansion relieves upper airway obstruction.

6. REM Sleep percentage
  - Assesses the proportion of sleep spent in REM, which can reflect improved sleep quality and architecture post-treatment.

##### Subjective Measures

1. ESS
  - A patient-reported questionnaire with eight questions that measures daytime sleepiness; a lower score post-treatment indicates improved alertness [3,41].

2. QSQ
  - A comprehensive, patient-reported tool designed to evaluate the QoL impact of OSA [3]. It consists of 32 items distributed across five domains: diurnal symptoms, nocturnal symptoms, daytime sleepiness, social interactions, and emotional impact [3].

3. NOSE Score
  - Patient-reported measure of nasal obstruction severity and its effect on QoL [42].

Collectively, the combination of objective metrics (AHI, oxygen saturation) and subjective assessments (ESS, QSQ) provides a comprehensive evaluation of treatment success, capturing both the physiological improvements and the patient-perceived benefits of maxillary expansion in OSA management.

### 6.5. Limitations, Risks, and Considerations

#### 6.5.1. Potential Risks and Complications

Complications reported following DOME are generally minor and manageable. Hypothetical risks of DOME include oronasal fistula, sinus infection, incisor tooth vitality loss, and paresthesia of the anterior maxillary division (V2) of the trigeminal nerve [27,36]. In the study by Liu et al. [7] involving 20 patients, no cases of malunion or tooth loss were reported; however, three patients exhibited minor asymmetric maxillary expansion, which was correctable with orthodontic intervention.

In a larger retrospective cohort of 75 subjects, no major adverse effects such as oronasal fistula, skeletal infection, nasal or sinus infection, malunion, or non-union were observed [27]. Paresthesia of the V2 nerve was noted, which resolved within 1–6 months [27]. Transient perfusion changes in maxillary central incisors were observed occasionally, which resulted in loss of vitality in 5% of patients [27]. However, root canal treatment was the treatment of choice, and there was no loss of dentition [27]. In 2% of the patients, periodontal attachment loss and incisional dehiscence were noted [27]. A palatal fistula was reported by a single patient, and it resolved without treatment [27]. Minor bone grafting was required by one patient [27]. The treatment protocol was adjusted in this study, and it was found that delaying orthodontic treatment after expansion reduced these complications noted above [27]. Like the earlier study by Liu et al. [7], this study also reported minor asymmetric maxillary expansion, also amenable to orthodontic correction [27].

In another retrospective study, no malocclusion or pain was noted, while five subjects (15.6%) experienced minor asymmetric expansion of the maxilla, which was manageable within orthodontic treatment [36]. These results appear consistent with the aforementioned studies. Paresthesia along the distribution of V2 in the anterior maxilla was reported in four patients (12.5%) and resolved within 6 months [36]. A single patient (3.1%) experienced incisor loss of vitality, and this was managed by endodontic treatment [36]. In a study evaluating DOME, none of the patients had cosmetic concerns post-expansion [36].

A retrospective study on DOME revealed no major adverse effects such as sinusitis, loss of teeth, malocclusion, malunion, or wound infection [37]. Consistent with the previous studies, 2 out of 32 patients had minimal asymmetric expansion of the maxilla, which was correctable with orthodontics [37]. The various studies examining the DOME technique revealed similar findings regarding adverse events, suggesting reproducibility and consistency in safety outcomes across studies.

#### 6.5.2. Variability in Treatment Response

Some variability in clinical outcomes has been noted. Iwasaki et al. [35] reported that two patients failed to demonstrate AHI improvement following DOME—one due to persistent nasal obstruction from severe septal deviation, and the other due to having the smallest pharyngeal airway volume in the cohort. These findings reinforce the importance of patient-specific anatomical and functional assessment prior to intervention.

#### 6.5.3. Postoperative Considerations and Counselling

Although DOME is less invasive than other surgical interventions, it still requires osteotomy under general anaesthesia, and therefore, potential associated risks must be acknowledged. Recovery time may vary depending on individual patient factors. Patients are typically instructed to follow a soft diet for two days postoperatively [36]. Although this is generally well tolerated, it might represent a minor inconvenience for some patients and must be discussed as part of preoperative counseling. Counseling should also address potential risks, such as the possibility of transient nerve symptoms and minor asymmetries. These factors should be clearly communicated to ensure informed consent.

### 6.6. Summary of Maxillary Expansion Techniques

Table 1 provides a summary comparing the four maxillary expansion techniques (RME, SARPE, MARPE, and DOME) in OSA.

## 7. Age-Specific Clinical Protocols for Maxillary Expansion

Throughout the stages of development, from infancy to adulthood, changes in the craniofacial region and dentition follow specific growth patterns, which can be intercepted and addressed at key developmental periods [43]. Timely intervention is critical for patients with sleep disorders to reduce the risk of long-term systemic complications [43]. In pediatric populations, periods of active somatic and craniofacial growth offer critical opportunities to apply tailored treatments [43]. Therefore, it is crucial that pediatric dentists and orthodontists are involved in the interdisciplinary management of children with sleep disordered breathing (SDB) and OSA. The observed link between SDB, OSA, and craniofacial disharmony is thought to stem from deviations in normal growth patterns [43]. As orthodontic treatments have the potential to influence and direct craniofacial development, age-specific clinical guidelines are necessary to ensure personalized and optimized care [43]. Since craniofacial growth carries on over time, orthodontists can influence its development by targeting specific anatomical structures and modifying growth trajectory when interventions are applied at the appropriate time [43].

### 7.1. Childhood and Early Adolescence Protocols (6–11 Years)

Age is not the only factor determining treatment modality, and other factors must also be considered. In a previous study, it was reported that an obese patient diagnosed with OSA showed a poor response to RPE [3]. This may be used to highlight the potential impact of obesity on hindering OSA resolution in patients with transverse maxillary deficiency. In pediatric patients with a normal BMI and OSA who failed to respond to adenotonsillectomy, RME may be considered as an alternative [3]. The positive effects of RME on upper airway dimensions are well-established in this age group, as it increases the minimal cross-sectional area and airway volumes [23]. The transverse deficiency of a hypoplastic maxilla is corrected with RME, achieving expansion at the nasal floor along the mid-palatal suture [27]. This results in decreased resistance to nasal airflow and a greater nasal cavity volume [27]. The tongue is able to protrude both forwards and upwards as a result of RME, increasing the volume of the posterior pharyngeal airway space during sleep [27].

- Childhood (6–9 years): Early Mixed Dentition: The recommended appliance for this age group is RPE since expansion is the easiest during this age due to minimal resistance from the mid-palatal suture [43]. The direction of nasomaxillary complex growth is primarily horizontal at this stage, and the response to expansion is favorable [43]. RPE is usually effective; however, if greater skeletal change is needed, TADs may be added for anchorage [43]. This is a useful treatment modality for children with narrow arches or airway concerns, especially those with adenotonsillar hypertrophy [43].
- Early Adolescence (10–11 years): Late Mixed Dentition: At this stage, transverse maxillary growth is largely complete, and further maxillary development occurs primarily in the vertical direction [43]. However, if the nasomaxillary complex still requires forward growth, MARPE may be a suitable option, and it can be used in conjunction with facemask therapy [43]. This allows forces from the mini-implants to be applied to the nasomaxillary complex, allowing forward midface skeletal development [43].

### 7.2. Middle Adolescence Protocols (12–15 Years)

*Permanent Dentition:* Non-invasive maxillary expansion could be achieved using dental expanders prior to suture fusion at approximately 15 years of age [7]. Patients under the age of 15 years with a transverse maxillary deficiency are treated with RME due to the gradual interdigitation of the mid-palatal suture with age [25]. Mid-palatal suture fusion typically occurs concurrent with the pubertal growth spurt [27]. In the cranial structure, growth is mostly completed at this stage. Peak growth, including that of the mandible and ramus, tends to occur between the ages of 13 and 15 years in boys and 11 and 13 years in girls [43]. Children and adolescents in whom the intermaxillary suture has not fully ossified are suitable candidates for RME [23]. In patients of this age with SDB, MARPE may still promote skeletal remodeling, although skeletal structures are approaching maturity and have surpassed their peak growth potential [43].

### 7.3. Late Adolescence Protocols (16–18 Years)

At this stage, most skeletal growth is complete, particularly in the craniofacial region, though residual mandibular and hyoid growth may continue. As patients reach skeletal maturity, treatment shifts towards direct skeletal interventions [43]. MARPE remains a viable option for managing high-arched narrow palates and transverse discrepancies if skeletal fusion is not complete [43]. In late adolescents approaching adulthood, MARPE remains an effective option for achieving suture split, particularly before the age of 20 [24]. It is effective for middle to late adolescents with maturing sutures who are past the peak growth period. If MARPE fails or the sutures are fully matured, DOME becomes an appropriate alternative [43]. SARPE may also be considered in patients over 18 years of age when surgical intervention is indicated.

### 7.4. Young Adults Protocols (19–25 Years)

RPE is associated with risks such as root resorption, tipping of posterior teeth, gingival recession, and relapse, especially in older patients [24]. MARPE enables maxillary expansion in young adults and adolescents by bypassing skeletal resistance without requiring surgery [43].

### 7.5. Adult Protocols (>25 Years)

A rapid increase in suture closure has been reported in patients from the age of 25 [24]. This may limit the effectiveness of non-surgical expansion techniques. RME is not typically used in adults due to mid-palatal suture ossification [23]. It may also cause adverse periodontal and dental effects such as dehiscence and bone thinning, specifically on the buccal aspect of the posterior dentition, along with buccal tipping of the molars [29].

In older adults, MARPE revealed inconsistent expansion outcomes due to reduced predictability of suture separation, even with the use of TADs [27]. Surgical intervention is typically required for skeletal changes and to treat moderate-to-severe OSA cases [43].

SARPE involves invasive Le Fort I osteotomies with fracture of the pterygoid plates and risks including non-union, malunion, and epistaxis [43]. Since SARPE is a surgical procedure, there is an increased cost, and hospitalization is required, along with absence from school or work [24].

Maxillary expansion in adult patients with OSA, a high arched narrow palate, and nasal obstruction could be tackled with DOME [7]. DOME offers consistent and reliable maxillary expansion without the unpredictability and complications of MARPE or SARPE [43]. DOME is the recommended technique as complete mid-palatal suture fusion is common in this age group, reducing the effectiveness of non-surgical expanders [43]. In DOME, the necessity for pterygoid disjunction is eliminated [7]. Not only is DOME less invasive, but minimal side effects are present in comparison to SARPE [27]. However, with DOME, the expanders need to remain in place at least for 8 months after the surgery. Studies on the relapse rate after DOME are not readily available. Advancements such as surgical osteotomy guides have enabled DOME treatment to become less invasive and performed with greater precision [43]. Some patients are reluctant to undergo surgical procedures; hence, DOME and SARPE would not be suitable options for such patients, and MARPE should be considered. Thus, patient preferences and individual factors such as age should be carefully considered to guide case selection.

## 8. Discussion

### 8.1. How Maxillary Expansion Complements Other OSA Treatments

The first line of OSA treatment is positive airway pressure (PAP), which remains the gold-standard treatment due to its high efficacy [44]. However, many patients struggle with tolerance or adherence, necessitating alternative or adjunctive treatments. Oral appliance therapy (OAT) is commonly used in mild to moderate OSA, or in combination with PAP for severe cases [44]. OAT includes devices such as mandibular advancement appliances (MAAs), which anteriorly displace the mandible and the surrounding soft tissues throughout sleep, reducing oropharyngeal airway collapse [44]. Another form of OAT is tongue retaining devices (TRDs), which hold the tongue in a forward position [44].

Additionally, maxillary expansion techniques may aid in the management of OSA in cases of a transverse maxillary discrepancy. Techniques for maxillary expansion include RME, SARPE, MARPE, and DOME, selected based on patient age, skeletal maturity, and severity of the transverse deficiency. Maxillary expansion increases nasal and oropharyngeal airway dimensions, improving upper airway patency and reducing resistance [45].

Treatment efficacy with PAP or oral appliance therapy is influenced by airway anatomy and pharyngeal stability [46]. These findings suggest that the anatomical improvements from maxillary expansion may support better outcomes when combined with PAP or OAT, based on the relationship between airway stability and treatment response. This complementary effect suggests that maxillary expansion could serve as an adjunct to conventional OSA management, particularly in patients with transverse maxillary deficiency. Combining maxillary expansion with PAP or oral appliances may improve outcomes, particularly in patients with transverse maxillary deficiency.

In adults with severe OSA and airway obstruction localized to the oropharynx or in the presence of maxillary and/or mandibular retrusion, maxillomandibular advancement (MMA) may be indicated [44]. However, MMA is generally unsuitable for patients with bimaxillary prognathism [44]. Overall, integrating maxillary expansion with established therapies offers a synergistic approach that addresses both anatomical and functional aspects of OSA.

### 8.2. Limitations of the Review

This review adds value by offering a comprehensive synthesis of current evidence on the impact of RME, SARPE, MARPE, and DOME on patients with OSA. While prior studies have often focused on individual techniques, this review compiles findings across a spectrum of skeletal expansion modalities, comparing their effects on airway volume, clinical outcomes, and suitability for different age groups.

Additionally, the review highlights mechanisms of action, patient selection criteria, side effects, and limitations, helping clinicians better understand the indications and outcomes associated with each approach. Unlike meta-analyses that may impose strict inclusion thresholds or focus on a narrow patient population, this review integrates findings across broader clinical contexts to assist in real-world decision-making.

By organizing diverse data into a structured, accessible format, this review serves as a practical reference for general dentists, orthodontists, sleep specialists, maxillofacial surgeons, and other professionals involved in the management of patients with OSA.

Despite the breadth of information synthesized in this review, several limitations must be acknowledged. First, as a narrative review, the methodology lacks the structured, reproducible protocols of systematic reviews or meta-analyses, which may introduce selection bias. No formal risk-of-bias tool was applied, and inclusion was based on relevance and availability of data, which could affect objectivity.

Second, there is heterogeneity across the included studies in terms of patient populations, diagnostic criteria, expansion protocols, and outcome measures. Moreover, variation in the tools and metrics used to evaluate outcomes—such as AHI, upper airway volume, cross-sectional area measurements, and subjective sleep questionnaires—complicates direct comparisons across studies and may influence perceived treatment efficacy. This variability limits the ability to draw direct comparisons or perform quantitative synthesis.

Although peer-reviewed sources were prioritized, publication bias remains a concern, as studies with positive findings are more likely to be published and accessed. Additionally, language restrictions and the 10-year publication window may have excluded potentially relevant older or non-English studies. Furthermore, many studies lack long-term follow-up, limiting our understanding of the durability of treatment effects across different expansion modalities. Finally, while the review covers four expansion techniques, direct head-to-head comparisons among them are limited in the literature, making it difficult to establish definitive clinical superiority of one modality over another.

Acknowledging these limitations allows for a more critical interpretation of the findings and underscores the need for future high-quality, comparative studies with standardized outcome reporting to strengthen the evidence base.

## 9. Conclusions

OSA is a prevalent condition with multiple available treatment modalities. In patients presenting with transverse maxillary discrepancy, expansion techniques such as RME, SARPE, MARPE, and DOME may be considered to alleviate upper airway obstruction and improve respiratory outcomes. Appropriate case selection is crucial to ensure successful treatment outcomes of expansion and, ultimately, OSA reduction. Future studies should investigate whether maxillary expansion techniques in growing and adult patients lead to long-term bone formation and how this contributes to the stability of skeletal and airway changes over time.

## Figures and Tables

**Table 1:** Comparison of maxillary expansion techniques (RME, SARPE, MARPE, and DOME).

	Technique	Advantages Over Other Techniques	Indications	Outcomes	Limitations/Complications
RME	An appliance applies lateral forces to the maxilla, opening the mid-palatal suture. Skeletal expansion occurs, and bone fills the suture separation.	No surgery is required Relatively non-invasive	Children and early-middle adolescents with transverse maxillary deficiency.	Allows proper alignment of teeth. Eliminates crossbites Enhanced breathing, speech, and overall facial aesthetics.	Pain and discomfort with temporary speech or eating difficulty during expansion. Does not correct complex skeletal discrepancies and additional treatment may be required. Relapse risk if retention protocols are not followed; a retainer is required.
SARPE	Requires general anesthetic for the surgical intervention. Combines selective osteotomies and mechanical expansion forces. Weakens resistance areas: mid-palatal suture, lateral nasal walls, pterygoid plates. Palatal appliance is gradually activated post-surgery; the maxilla splits, and new bone fills the expansion gap.	Provides true skeletal expansion in skeletally mature patients. Reduces dental tipping and alveolar bending risks.	Skeletally mature patients with transverse maxillary deficiency. Cases where non-surgical expansion fails. Prepares space for dental alignment, orthognathic surgery, and improves smile esthetics and function.	Dependable skeletal expansion. Expands nasal airway, improves breathing and chewing function. Enhanced facial esthetics and overall QoL.	More invasive and costly. Surgical risks: infection, bleeding, nerve injury, swelling, discomfort. Risk of harm to the nasal mucosal tissues and tooth roots. Post-operative discomfort and temporary functional limits. Requires strict retention; poor compliance may lead to relapse or asymmetry. Precise planning required for the surgical process and liaising of the orthodontist and surgeon.
MARPE	No osteotomies required. Expander placed intraorally on basal bones under local anesthesia. Mini-implants inserted into pre-perforated palatal bone using a bur. Active expansion for 4–6 weeks, followed by a retention period. Mid-palatal suture opens with lateral displacement of the nasal walls.	Avoids dental anchorage, minimizing root resorption and periodontal defects. Greater skeletal expansion in comparison to RME. Non-surgical alternative for improving airway function in adults.	Transverse maxillary deficiency in late adolescents and young adults. Success influenced by suture ossification stage.	Reduces nasal resistance and enhances airway patency. Improves breathing during sleep. Increase in nasopharyngeal airway volume and nasal cavity volume.	Retention required to prevent relapse. Soft tissue irritation, inflammation, tenderness, or pain around the appliance during and after expansion. Risk of appliance breakage, asymmetric expansion, or minor papillary recession. Rare complications: short-term hearing loss, swelling, numbness, paresthesia, sinus infection, severe gag reflex, discoloration of teeth, possible loss of vitality, infraorbital nerve hypoesthesia. Treatment success depends on patient compliance and the stage of suture maturation; success declines with age.
DOME	Maxillary expander is secured with mini-implants; minimally invasive Le Fort I osteotomy under general anesthesia Expander activated 5–7 days post-surgery, daily for approximately one month. Gap heals naturally with bone regeneration.	Less invasive osteotomy compared to SARPE. Minimizes dental tipping.	Adults with OSA and a transverse maxillary deficiency. High-arched palate, minimal soft tissue excess. Cases requiring enhanced airway volume and functional improvement. Late adolescents with failed MARPE or fully mature sutures.	Expands maxilla and nasal floor, reducing airway resistance. Substantial AHI reduction and improved oxygen saturations. Enhanced REM sleep, NOSE and ESS scores and radiographic airway widening. Overall functional airway and QoL improvement.	Requires general anesthesia with surgical risks (infection, bleeding, nerve injury). Loss of vitality of maxillary incisors. Risk of oronasal fistula, sinus infection, asymmetric expansion or malunion. Temporary or permanent paresthesia of the anterior maxilla possible; 6–8 months of retention required to ensure bone fill and prevent relapse.

## Data Availability

No new data were created or analyzed in this study. Data sharing is not applicable to this article.

## Abbreviations

The following abbreviations are used in this manuscript:

OSA	Obstructive Sleep Apnea
QoL	Quality of Life
RME	Rapid Maxillary Expansion
MARPE	Mini-screw-Assisted Rapid Palatal Expansion
SARPE	Surgically-Assisted Rapid Palatal Expansion
DOME	Distraction Osteogenesis Maxillary Expansion
NC	Neck Circumference
CBCT	Cone-beam Computed Tomography
AHI	Apnea-hypopnea Index
RPE	Rapid Palatal Expander
TB	Tooth-borne
TBB	Tooth-bone-borne
BB	Bone-borne
TAD	Temporary Anchorage Device
ESS	Epworth Sleepiness Scale
QSQ	Quebec Sleep Questionnaire
CT	Computed Tomography
BMI	Body Mass Index
ODI	Oxygen Desaturation Index
NOSE	Nasal Obstruction Symptom Evaluation
REM	Rapid Eye Movement
SBD	Sleep Disordered Breathing
PAP	Positive Airway Pressure
OAT	Oral Appliance Therapy
MAA	Mandibular Advancement Appliances
TRD	Tongue Retaining Devices
MMA	Maxillomandibular Advancement
